# Continuous decoding of grasping tasks for a prospective implantable cortical neuroprosthesis

**DOI:** 10.1186/1743-0003-9-84

**Published:** 2012-11-26

**Authors:** Jacopo Carpaneto, Vassilis Raos, Maria A Umiltà, Leonardo Fogassi, Akira Murata, Vittorio Gallese, Silvestro Micera

**Affiliations:** 1 Neural Engineering Area, The BioRobotics Institute, Scuola Superiore Sant’Anna, Pisa, Italy; 2 Department of Basic Sciences, Faculty of Medicine, School of Health Sciences, University of Crete, Iraklion, Greece; 3 Foundation for Research and Technology-Hellas, Institute of Applied and Computational Mathematics, Iraklion, Greece; 4 Department of Neuroscience, Section of Physiology, University of Parma, Parma, Italy; 5 Italian Institute of Technology, RTM, Parma, Italy; 6 Department of Psychology, University of Parma, Parma, Italy; 7 Department of Physiology, Kinki University Faculty of Medicine, Osaka-Sayama, Japan; 8 Translational Neural Engineering Lab, Center for Neuroprosthetics and Institute of Bioengineering, Ecole Polytechnique Federale de Lausanne, Lausanne, Switzerland

**Keywords:** Ventral premotor cortex (area F5), Brain decoding, Grasping, Neuroprosthesis

## Abstract

**Background:**

In the recent past several invasive cortical neuroprostheses have been developed. Signals recorded from the motor cortex (area MI) have been decoded and used to control computer cursors and robotic devices. Nevertheless, few attempts have been carried out to predict different grips.

A Support Vector Machines (SVMs) classifier has been trained for a continuous decoding of four/six grip types using signals recorded in two monkeys from motor neurons of the ventral premotor cortex (area F5) during a reach-to-grasp task.

**Findings:**

The results showed that four/six grip types could be extracted with classification accuracy higher than 96% using window width of 75–150 ms.

**Conclusions:**

These results open new and promising possibilities for the development of invasive cortical neural prostheses for the control of reaching and grasping.

## Findings

### Introduction

In the recent past many efforts have been devoted to develop artificial devices to restore sensorimotor functions in people who lost them due to amputation, spinal cord injury, stroke, etc. [1-3]. The possibility of connecting the peripheral and central nervous system with artificial devices by means of invasive neural interfaces [1-5] is currently investigated in order to increase the number of possible functional connections between patients with impaired sensory/motor functions and the artificial device (e.g., hand prostheses, robotic arm, etc.) and to control it in a simple and intuitive way. Electrodes have been implanted invasively a) in peripheral nerves to achieve a bi-directional control of hand prostheses in amputees [6,7]; b) in the cortex, to extract user’s motor commands from movement-related cortical signals [2,8,9] or to deliver a sensory feedback by stimulating selected sectors of the somatosensory cortex [10]. Thus, invasive cortical neural prostheses (ICNPs) could help subjects affected by several deficits caused by spinal cord injury, stroke, amyotrophic lateral sclerosis, cerebral palsy, and multiple sclerosis to re-establish some degree of autonomy by controlling an output on a computer or a robotic system. In most cases, research groups have focused their efforts on the extraction of information from the motor cortex (area M1) to drive a robotic arm: signals recorded from ensembles of M1 cortical neurons have been processed through different algorithms to predict reaching directions or trajectories of a robotic arm end-effector [11-14]. This approach has been tested with very promising results in animal models [2,12] and recently in selected highly disabled subjects [8,9,15]. Moreover, individual [16] or ensemble M1 neurons data [17] have been used in order to predict hand or forearm muscle activity in brain controlled functional electrical stimulation (FES).

The situation becomes more challenging when several degrees of freedom need to be controlled (e.g., dexterous hand prostheses). In this case, even if recent results have shown that information related to finger movements can be obtained from M1 activities [18,19], it seems quite difficult to extract simultaneously the kinematics of all the fingers using this kind of approach. Therefore, the possibility of decoding higher level information (e.g., the specific grip type instead of the trajectories of the hand joints) may offer several advantages. Previous experiments have shown that neurons of ventral premotor area F5 (located in the posterior bank of the inferior limb of the arcuate sulcus and the cortical convexity immediately adjacent to it) do not specify/encode a given pattern of movements (as M1 neurons), but rather an end-state, like the goal of a motor act (e.g., grasping as a whole) [20]. Motor neural activity, recorded using single microelectrode [21], multi electrode array [22], ECoG [23], and Local Field Potentials [24], changes according to the properties of the object to be handled and, thus, of the grasping task. Recent studies demonstrated that it is possible to distinguish between power vs. precision grip [22,23] or up to six grips considering different duration periods of the whole movement phase [25]. Starting from this neurophysiological rationale, a pattern recognition algorithm for a continuous decoding of four/six grip types from F5 neurons during execution of a reach-to-grasp task is presented in this manuscript.

### Materials and methods

Data analyzed in this paper have been collected from area F5 in the posterior bank of the inferior limb of the arcuate sulcus in three hemispheres (contralateral to the moving forelimb) of two awake monkeys (Macaca nemestrina). Behavioral apparatus and task, animals training and data collection have been described in [21]. Briefly, the monkey seated in front of a rotating turntable subdivided into six sectors, each containing a different object. The monkey had to fixate one object and press a key then release it, reach for and grasp the object, pull it, hold it and release it. The different objects were presented to the monkey in random order (8 repetitions for each object). Two sets of six geometric objects eliciting different grip types were used (i.e., *original* and *special* set composed by objects differing in size and shape, see Table 1 and [21]).

**Table 1:** **The objects of the original and special set and the grips used by the monkeys during grasping**[21,25]

**Original set**	**Grip type**	**#**	**Special set**	**Grip type**
Cube	Side grip	1	Sphere in groove	Advanced precision grip
Sphere	Side grip	2	Large cylinder in container	Finger prehension with thumb opposition
Cone	Side grip	3	Small sphere	Side grip
Plate	Primitive precision grip	4	Large sphere	Whole hand prehension
Cylinder	Finger prehension	5	Small ring	Hook grip (index)
Ring	Hook grip (index)	6	Large ring	Hook grip (4 fingers)

The dataset used in this paper consisted of 46 F5 neurons that were classified as purely motor grasping neurons. The activity of these neurons was not related to individual finger movements, but to the grasping action as a whole [21]. Thirty-six were tested with the objects of the original turntable and 10 with the objects of the special turntable. Properties of the neurons have been analyzed in [21] whereas the results of grips classification during different duration periods of the movement phase have been reported in [25].

#### Decoding algorithm

In order to investigate whether a reliable grip classification can be obtained during the object presentation and movement phase, normalized firing rate (nFR) have been extracted from F5 motor units during a period that goes from 1800 ms before to 200 ms after the key release event (e.g., starting of the movement phase). In particular, nFR was extracted for each unit of the F5 population and in each trial using analysis windows varying in duration (bin widths from 25 to 200 ms) which progressively slid over the reference period with a moving step of 10 ms. Normalization was done with respect to the maximum nFR among all trials. The F5 neural population response onset (t_onset_) has been detected using a threshold algorithm [26]. The onset corresponded to the moment when the F5 population nFR in an analysis window was more than 2.5 of standard deviation value, calculated over the first 400 ms of the object presentation phase (from −1800 to −1400 ms). The sub-threshold activity before key release (object presentation) was considered as baseline. The baseline and the above-threshold activity recorded during execution of the different grips were labeled and used as examples to train the classifier or to test its generalization skills. nFR of the different neurons have been classified using SVMs (ν-SVMs with radial basis kernel function) making use of the open source library LIBSVM [27]. Training and cross-validation has been done splitting data by trials and using a random selection of 25% of the trials for the training of the classifier and the remaining 75% of the trials for the testing. Two different classification methods have been used: i) direct discrimination of 5/7 classes (baseline plus four/six grip types); and ii) hierarchical discrimination (baseline versus above-threshold activity and then selection of four/six grip types). In the case of four objects classification, the first three object of the original set (i.e., cube, sphere, and cone) as well as the first three object of the special set (sphere in groove, large cylinder in container, and small sphere) have been clustered together because the grips used for their prehension shared common features (i.e., side grip or thumb/finger opposition) [21,25]. The accuracy of the objects classification has been assessed using a recognition ratio (RR), defined as the proportion of the grips correctly identified with respect to those classified.

#### Statistical analysis

A Friedman test (p ≤ 0.01) has been used to compare the results obtained from (i) the neurons tested with the two sets of objects (original vs special) and (ii) the two classification schemes (direct vs hierarchical discrimination). Moreover, a Kruskall-Wallis test (p ≤ 0.01) has been used to verify the influence of the different bin-widths in the classification accuracy.

### Results

**Figure 1:**
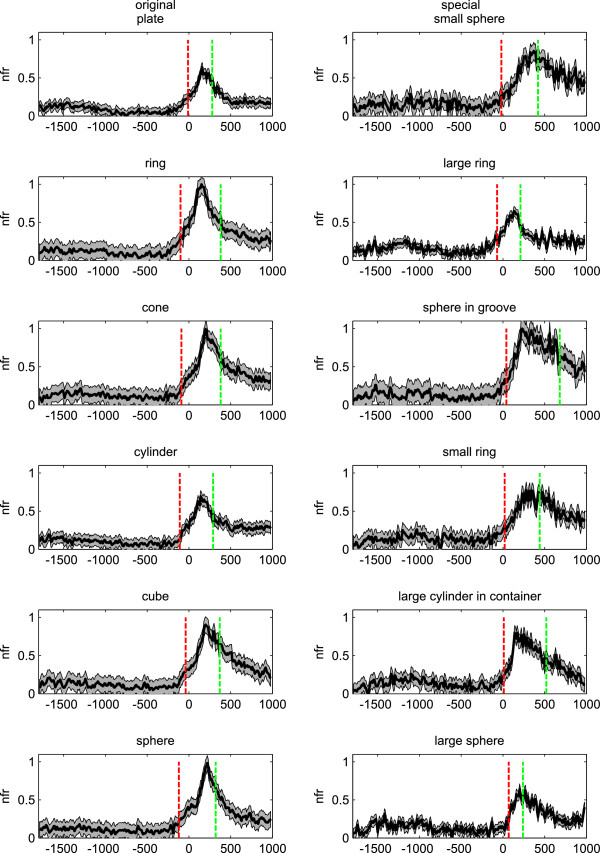
**The mean normalized firing rate (nFR) ± standard deviation, during grasping of different objects, of the F5 neurons tested with the original (left) and the special (right) set of objects (bin width = 25 ms).** t = 0 corresponds to the key release event (start of the movement phase). The mean t_onset_ and t_mov_ for each object are indicated by red and green vertical lines.

In Figure 1, the mean nFR of the F5 motor neurons population for each object of the normal and special set is given. The response has been plotted from 1800 ms before to 1000 ms after the onset of the movement (the key release event that corresponds to 0 ms). The key release event corresponds to 0 ms). The mean t_onset_ and the mean end of the movement (t_mov_) are marked as red and green horizontal line, respectively.

In the case of the original set of objects, the onsets of the neural population response, calculated with a threshold method (see Materials and methods section), preceded the start of movement whereas, in the case of special set of objects, the onsets were either preceding or following the start of the movement. t_mov_ was 334 ± 113 ms for the original set of objects and 407 ± 192 ms for the special set of objects. In both cases, the prediction of grip type was done in the first 200 ms of the movement phase well before its end.

**Figure 2:**
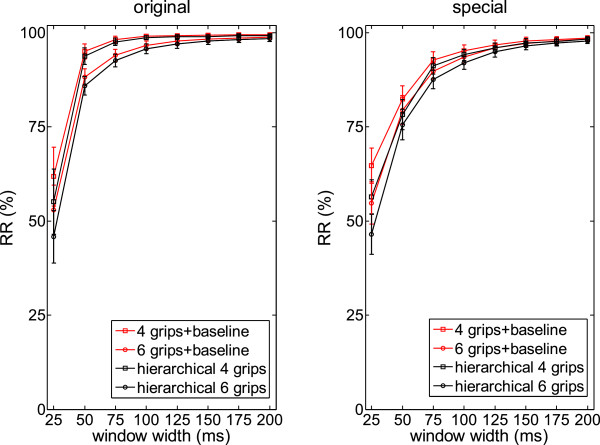
**Performance of the SVM classifier as a function of the number of grips to be recognized (RR = recognition ratio).** Left panel: original set; right panel: special set.

The results of the classification as a function of the window width (from 25 to 200 ms) are given in Figure 2 for all the different classification analyses (four or six grips, direct or hierarchical, original or special set).

Window widths between 75–150 ms seem to be sufficient to obtain the highest values of stable RR. More specifically, with the original set of objects and a window widths of 100 ms, RR values were 99.11 ± 0.52 (direct) and 98.70 ± 1.16 (hierarchical) for the classification of 4 grips and 96.62 ± 1.16 (direct) and 95.74 ± 1.32 (hierarchical) for the classification of 6 grips. With the special set of objects and a window width of 150 ms, RR values were 97.79 ± 1.01 (direct) and 97.30 ± 1.17 (hierarchical) for the classification of 4 grips and 97.18 ± 0.95 (direct) and 96.51 ± 1.09 (hierarchical) for the classification of 6 grips.

The performance of the two populations of neurons was significantly different (p < 0.01) and this is likely due to the number of neurons belonging to each population (i.e., 36 and 10 neurons tested with the original and the special set of objects, respectively). Nevertheless, the differences between the mean accuracy obtained from the neurons tested with the original and special set of objects were limited (direct classification of 4 and 6 grips: 3.14% and 2.31%, respectively; hierarchical classification of 4 and 6 grips: 4.03% and 2.88%, respectively). These differences decreased below 2% using bins greater than 150 ms. Concerning the classification schemes, even if the direct approach was significantly better than the hierarchical one (p < 0.01), the differences between the two schemes were less than 2% (1% using window width greater than 125 ms). Finally, window length influenced significantly the accuracy (p < 0.01). The use of small bins (25 ms) resulted in deteriorated performance (i.e., differences worse than 30% as compared to the mean accuracy obtained with the use of 100 ms bins) whereas the use of bins larger than 150 ms did not result in any significant improvement (i.e., 0.34% and 0.72% differences in the mean accuracy between bins of 150 and 200 ms with the original and the special set of objects, respectively).

**Figure 3:**
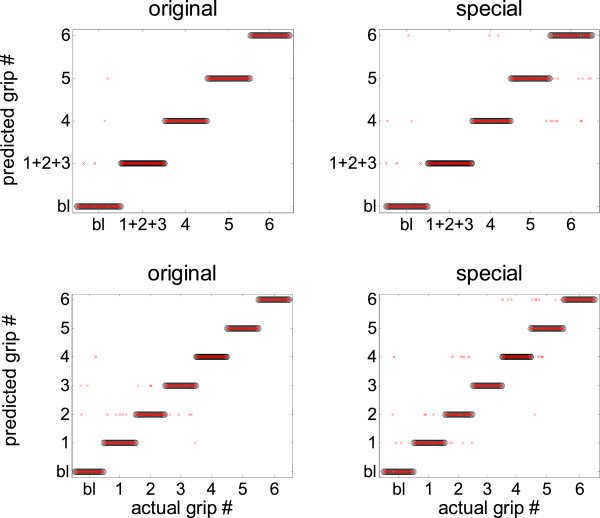
**Direct discrimination of 5/7 classes simultaneously (baseline plus four/six grip types) using a window width of 100 ms.** Grip numbers according to Table 1, bl = baseline. X axis: actual grips (sorted). Y axis: grips predicted by the classifier. Correct classification results are shown as superimposition between the actual grip (black ovals) and predicted grip (red stripes). Classification errors are shown as isolated red crosses.

An example of the classification accuracy during the direct discrimination of 5/7 classes obtained with a 100 ms window width is given in Figure 3.

Actual grips are represented by black ovals whereas predicted grips are represented by red crosses. Correct classification results in a superimposition between actual and predicted grips (i.e., black ovals and red stripes). Isolated red crosses represent errors made by the classifier and are plotted at the level of the predicted class. Most of the errors occur during the classification of 6 grips. In the case of the original set, the classifier assigns part of the features extracted during the grasping of objects 1 and 3 to grip 2, confirming the fact that objects 1, 2, and 3 are grasped in a similar way [21]. For the special set of objects, a similar behavior occurs for objects 1 and 2 and for the objects 5 and 6 (hook grip) [21].

### Discussion

**Figure 4:**
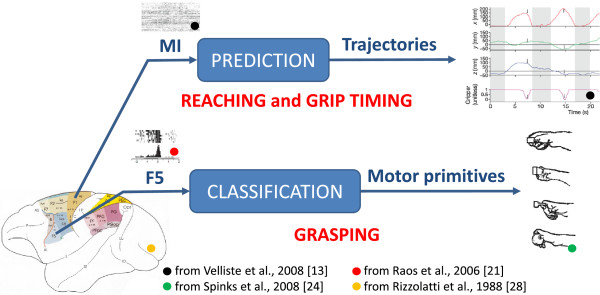
**A possible approach for the control of ICNP based on the combination of information about reaching and timing from M1 together with information about the grip type from F5. **Black circle from [13], red circle from [21], green circle from [24], and yellow circle from [28].

The extraction of grip types (e.g., precision grip, finger prehension, whole hand prehension) from F5 could be a very attractive solution to be applied complementary to the M1-based ICNPs. In fact, if the goal of the ICNP is to control both reaching and manipulation of a dexterous arm-hand artificial robot, signals recorded from M1 can be used to decode information about the reaching phase and grasp timing (as already proved by the very interesting results achieved so far [13,14]) while F5 signals can be used for the detection of the desired grasping task (see Figure 4).

Recent papers [22,23] demonstrated the possibility to distinguish between precision and power grips in a reliable way whereas in [25] the decoding of 4–6 grips during the reaching phase has been analyzed in different normalized windows (e.g., from 25% to 100% of the reaching phase).

In this paper, an SVM classifier has been used to continuously predict different grips (4 and 6) from the activity of F5 motor neurons recorded during the reach to grasp task. This classifier was already used in a previous paper from our group for the decoding of different grips [25]. However, in the current paper a population of purely motor neurons was analyzed whereas in [25] a population of motor and visuomotor units was employed. Secondly, in the present paper we investigated the effect of using different time windows while in the previous work one fixed window was used. As shown in Figure 1, activity of F5 neurons precedes or is contemporaneous to the onset of the movement. During hand transport phase and using a continuous classification scheme, it is possible to predict four/six grips with high RR using firing rate activity calculated over bins of 75 ms (for the original set of objects) and 150 ms (for the special set of objects). These results seem to be compatible with a real-time control of manipulation tasks (i.e., reaching and grasping) performed with ICNPs. In fact, the delay introduced by the grip decoding is similar to the maximum values proposed in case of an EMG-based control of prostheses (e.g., 100–125 ms [29]). motor related discharge, may allow the correct discrimination among grips even earlier than that reported in the present study [21,25].

It must be taken into account that the decoding algorithm has been tested offline with single cell recordings. Real-time ICNPs with chronic multi-electrode arrays may perform worse, even if this limitation could be reduced thanks to the progress of micro and nanotechnologies [4] and the learning-induced tuning of the cells during real-time experiments [30]. Another issue is the limited number of decoded grips. Nevertheless, four or six grips should ensure a good grasping dexterity and a significant increase in the number of possible activities of daily living to be carried out.

In conclusion, a SVM based algorithm has been used for a continuous decoding of grip types from F5 motor neurons during execution of reach-to-grasp tasks. The results obtained show that four/six grip types were extracted with classification accuracy higher than 96% using window width of 75–150 ms. These results introduce new and promising scenarios for the development of ICNPs. In fact, the possibility not only to control computer cursors and robotic devices but also to select actions or grips could represent a real improvement of functionality for neuroprosthetic devices.

## Abbreviations

ICNPs: Invasive cortical neuroprostheses; M1: Primary motor cortex; nFR: Normalized firing rate; RR: Recognition ratio; SVMs: Support vector machines.

## Competing interests

The authors declare that they have no competing interests.

## Authors’ contributions

JC and SM developed the decoding algorithm. VR, MAU, LF, AM, and VG designed the experimental protocol and performed the animal experiments. All authors wrote, read and approved the final manuscript.
